# The Story of the Insane from Year to Year

**Published:** 1904-07-30

**Authors:** 


### THE STORY OF THE INSANE FROM YEAR TO YEAR.

SIXTEENTH ANNUAL SERIES.

### LONDON COUNTY ASYLUM AT BANSTEAD.

ON the first day of January this asylum contained 2,457 patients and at the end of the year the numbers had fallen to 2,445. There were 440 first admissions, 63 readmissions, 210 recoveries, 31 discharged relieved, 22 discharged not improved, and 252 deaths. The total number under treatment during the year was 2,960, and the average daily number resident was 2,453. The recoveries were 43·57 per cent. of the admissions, and the deaths were 10·27 per cent. of the average number resident. Both of these percentages are somewhat higher than the average. Of the year's deaths 111 were caused by cerebral and spinal diseases, general paralysis alone accounting for 68 of that number, consumption was the cause in 44, pneumonia in 14, enteritis in 3, and small-pox in 2. In spite of all precautions taken to prevent the introduction of the latter disease when it was prevalent in London it appeared simultaneously in two female patients who were in the infirmary ward; and altogether 12 cases occurred. Of course they were immediately isolated in the detached hospital, and we think Dr. Johnston Jones is to be congratulated that he escaped so easily; but he must have had an anxious, trying time for awhile. The committee of visitors placed on record, by special resolution, their appreciation of "the admirable manner in which the medical superintendent coped with the difficulty." Of the year's admissions 52 were supposed to owe their insanity to mental anxiety and other moral causes, and among the physical causes hereditary influence accounts for 52, previous attacks for 104, and intemperance in drink for 83. The terrible fire at the Colney Hatch Asylum had one good effect: it induced committees and officials all over England to increase their preventives against the occurrence of fire and their means of coping with a fire should one unhappily break out. At Banstead the committee applied for leave to substitute permanent buildings for the temporary ones now in use, but the Commissioners very properly declined to sanction any proposal which would allow more than 2,000 patients in any asylum. We can only regret that this sensible resolution was not taken by the Commissioners a great many years ago, and that 1,000 was not the limit; but it is better late than never, and half a loaf is better than no bread at all. England may well be proud of her county asylums, speaking generally, but this hoarding together such vast numbers of the insane is quite inexcusable. It makes supervision and individual knowledge of patients well-nigh impossible, it necessitates a great many highly-paid officials, and it is expensive to the ratepayers. Although Dr. Johnston Jones points out that consumption was the cause of death in 22 per cent. of the male deaths, and 14 per cent. of the female deaths, he does not say how many cases he had during the year, nor whether anything is likely to be done to separate them from the others. Dr. Passmore, one of the assistant medical officers, has been appointed medical superintendent of the new asylum at Croydon. We congratulate him on his promotion, and also the assistant medical officers who got "steps up," as a consequence of his appointment. The weekly cost per head was nearly 10s. 7d.

